# Ethylene oxide as an occupational contact allergen – an underestimated problem? 

**DOI:** 10.5414/ALX01311E

**Published:** 2017-08-04

**Authors:** K. Breuer, M. Worm, C. Skudlik, S.M. John

**Affiliations:** 1 Dermatologikum Hamburg, Germany,; 2 Allergy-Centrum Charité Campus Mitte, Universitätsmedizin Berlin, Department of Dermatology and Allergy, Berlin, Germany,; 3 Institute for Interdisciplinary Dermatological Prevention and Rehabilitation (iDerm), University of Osnabrück, Germany, and; 4 Department of Dermatology and Environmental Medicine, University of Osnabrück, Germany

**Keywords:** ethylene oxide, epichlorohydrin, contact allergy, occupational contact dermatitis, irritant contact dermatitis, patch test

## Abstract

Background: Ethylene oxide (EtO) is a volatile epoxy compound which is used to sterilize medical devices. EtO may cause irritant contact dermatitis, but only few cases of allergic contact dermatitis have been reported yet. Objectives: About 20 employees of a department for surgery developed eczematous skin reactions at the contact areas to wrist bands of surgical gowns which had been sterilized with EtO. Patch tests were performed to exclude contact allergy. Methods: Due to the volatility of EtO, patch tests were done with epichlorohydrin (0.1% pet., 1% pet.) which is an epoxy compound chemically related to EtO. Results: 7/8 patients and 4 healthy control persons showed non-allergic irritant reactions to 1.0% epichlorohydrin. 1.0% epichlorohydrin may have induced an iatrogenic sensitization in one of the control persons. None of the control persons reacted to 0.1% epichlorohydrin. Allergic contact dermatitis to EtO and a cross sensitization to epichlorohydrin was diagnosed in a nurse who showed an allergic crescendo patch test reaction to 0.1% epichlorohydrin. Conclusions: EtO can act as an occupational contact allergen in health personnel, a problem that may have been underestimated in the past due to methodological difficulties in patch testing. When allergic contact dermatitis to EtO is suspected, a patch test to 0.1% epichlorohydrin should be performed.

German version published in Allergologie, Vol. 33, No. 8/2010, pp. 331-336

Ethylene oxide (EtO) is a gas which is widely used for the sterilization of medical supplies, especially heat-unstable materials such as textiles; it is known to be a strong irritant [1]. Recently, we were contacted by a large department for trauma surgery since about 20 employees had developed eczematous skin reactions at the contact areas to wrist bands of surgical gowns which had been sterilized with EtO, and contact allergy was suspected. Due to the volatility of EtO, patch tests were performed with epichlorohydrin, a substance chemically similar to EtO, as described lately [2]. Since recommendations for the adequate epichlorohydrin patch test concentration are not consistent [2, 3, 4, 5, 6], 0.1% and 1.0% epichlorohydrin were used in order to identify an appropriate patch test method. 

## Patients and methods 

In all 20 employees the eczematous lesions persisted for several weeks and faded only slowly after EtO-sterilized gowns had been replaced by gamma-irradiated ones. In some cases topical or systemic corticosteroids had been used for the treatment of eczema. 8 employees who had experienced contact dermatitis after the use of EtO-sterilized gowns (6 surgical (male) nurses, 2 surgeons) (Table 1) and 4 healthy controls from our department gave informed consent to a patch test. Patch tests were performed on healthy back skin. Contact dermatitis had cleared before the patch test was applied. 

Epichlorohydrin was obtained from Sigma-Aldrich, Taufkirchen, Germany. Patch tests with epichlorohydrin (0.1% pet., 1% pet.) were performed according to the guidelines of the German Contact Dermatitis Research Group (DKG) using Hayes Test Chambers (Hayes Service, The Netherlands). Moreover, wrist band samples of EtO-sterilized and gamma-irradiated surgical gowns moistened with 0.9% sodium chloride solution were patch tested on back skin after tape stripping. Evaluation was carried out at Day 1 (24 hours after application), Day 2 and Day 3. 

## Results 

No allergic reactions to the wrist bands of EtO- and gamma-irradiated gowns were observed. One patient (Patient No. 8 (Table 1)) exhibited an erythema at the patch test site of the EtO-sterilized gown at Day 1 which was rated as a non-allergic irritant reaction. 

**Figure 1.**
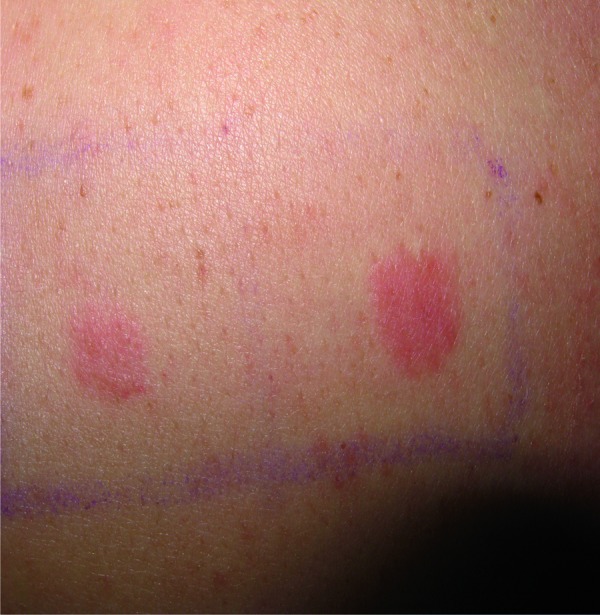
Allergic patch test reactions to epichlorohydrin 0.1% and 1.0% in a surgical nurse with allergic contact dermatitis to ethylene oxide.

3 patients showed at least an erythematous reaction to 0.1% epichlorohydrin (Patients 1 – 3 (Table 1)). A surgical nurse (Patient No. 1) developed a crescendo reaction with an erythema and an infiltration at Day 3 which was classified as an allergic patch test reaction (Figure 1). This patient was not available for the Day 2 reading. In Patient No. 2, we observed a decrescendo reaction to 0.1% epichlorohydrin with an erythema and an infiltration at Day 2 which had faded at Day 3. Patient No. 3 showed an erythema at Day 1. Patients Nos. 4 – 8 did not show any reactions to epichlorohydrin 0.1%. 

All employees showed at least an erythematous reaction to 1.0% epichlorohydrin. Again, Patient No. 1 exhibited a crescendo patch test reaction with an erythema and an infiltration at Day 3. Patients Nos. 2 and 4 exhibited an erythema and an infiltration at the patch test site of epichlorohydrin 1.0% in the Day 1, Day 2 and Day 3 readings. In Patient No. 3, we observed a bullous reaction to 1.0% epichlorohydrin at Day 1, Day 2 and Day 3. Patient No. 5 showed an erythema and an infiltration in the Day 2 reading to 1.0% epichlorohydrin with a decrescendo phenomenon at Day 3. Patients Nos. 6 – 8 exhibited an erythema to 1.0% epichlorohydrin in the Day 1, Day 2 and Day 3 readings. 

4 healthy control persons were also patch tested with 0.1% and 1.0% epichlorohydrin. Neither an erythema nor an infiltration was observed upon patch testing with 0.1% epichlorohydrin in the Day 1 – Day 3 readings. All individuals showed erythematous patch test reactions to 1.0% epichlorohydrin at Day 1, which were rated as irritant reactions. Therefore, the patch test reactions to 1.0% in Patients Nos. 2 – 8 were judged as non-allergic, irritant reactions. 

One of the controls exhibited a bullous irritant patch test reaction to 1.0% epichlorohydrin at Day 2 and Day 3. At Day 8, erythema and infiltration had developed at both the 0.1% and 1.0% epichlorohydrin patch test sites, which may point to an iatrogenic sensitization. 

According to the patch test results, contact allergy to EtO and a cross sensitization to epichlorohydrin was diagnosed in Patient No. 1 who showed a crescendo patch test reaction to 0.1% and 1.0% epichlorohydrin. This patient had experienced eczematous lesions at the contact sites with an EtO-sterilized gown spreading to the volar aspects of the forearms. She had a history of latex allergy and contact allergy to thiuram. Since she had worn vinyl gloves at work, contact dermatitis to latex or additives of rubber gloves is not probable, moreover, her contact dermatitis had firstly been localized at the wrists and the hands were not involved. 

Irritant contact dermatitis to EtO was diagnosed in Patients Nos. 2 – 8 who showed irritant reactions to epichlorohydrin. In these patients, eczema had been limited to the contact sites with the wrist bands of the EtO-sterilized gowns. 

## Discussion 

**Figure 2.**
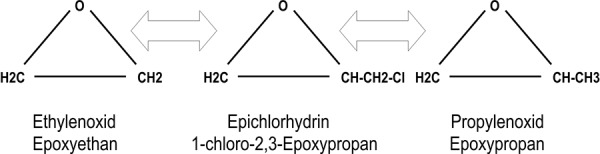
Chemical structure of ethylene oxide, propylene oxide and epichlorohydrin.

Here we report on a series of occupational contact dermatitis to EtO in health care personnel. Allergic contact dermatitis to EtO was diagnosed in a surgical nurse (Patient No. 1) by patch testing with epichlorohydrin, whereas irritant contact dermatitis was diagnosed in the other employees. Due to the volatility of EtO, no standardized patch test substance for EtO does exist. Morris and colleagues [4] reported a case of occupational allergic contact dermatitis to the solvent epoxy propane in an electron microscopy technician. Epoxy propane (propylene oxide) is an epoxy compound chemically similar to EtO with one extra methyl group. In this patient, patch tests were performed with both epoxy propane and epichlorohydrin (each 1.0%), which is used as raw material in the production of bisphenol A based epoxy resins. Epichlorohydrin differs from epoxy propane by only one chlorine atom (Figure 2). Both compounds gave positive results which points to a cross reaction between these substances. Recently, Kerre and Goossens [2] reported a nurse who developed contact dermatitis to an EtO sterilized gown and showed a positive patch test reaction to 1.0% epichlorohydrin, which is also chemically similar to EtO (Figure 2). 

In our observation, 1.0% epichlorohydrin yielded irritant patch test reactions in 7/8 patients and in 4 healthy control persons. Moreover, an iatrogenic sensitization to 1.0% epichlorohydrin may have occurred in 1 of our controls [7]. No control individual reacted to 0.1% epichlorohydrin, which suggests that 0.1% is an appropriate patch test concentration [3]. It is uncertain whether Kerre and Goossens have performed patch tests with 1.0% epichlorohydrin also in healthy controls. However, the use of 1.0% epichlorohydrin for the confirmation of contact allergy has also been reported earlier [4, 5, 6]. Patch tests with samples of EtO sterilized gowns did not lead to patch test reactions in our patient with allergic contact dermatitis to EtO. Materials such as fabrics which rapidly lose EtO, are known to produce few patch test reactions even with high levels of EtO [1]. Moreover, the employees had worn surgical gloves over the wrist bands and the occlusive milieu with heat and sweating at the wrists may have promoted the development of contact dermatitis under work conditions. 

Allergic reactions to ethylene oxide have been reported in the past and they mostly have been classified as IgE-dependent immediate type hypersensitivity reactions. Patients undergoing dialysis become sensitized to EtO due to repetitive contact [8, 9]. Furthermore, occupational asthma due to immediate type hypersensitivity to EtO after the use of latex gloves sterilized by ethylene oxide has been described [10]. 

Few cases of delayed type hypersensitivity reactions to ethylene oxide have been reported. A case of allergic contact dermatitis to EtO-sterilized suture material occurred in a patient who developed inflammatory lesions 8 days after a skin biopsy had been performed and who experienced a systemic reaction with fever, lumbar pain and malaise. A provocation test with a stitch using EtO-sterilized suture material resulted in an erythematous plaque whereas a stitch with gamma-radiation sterilized suture material did not lead to any skin reaction [11]. The inflammatory plaque histology showed a granulomatous reaction. Occupational allergic contact dermatitis to EtO has been described in health personnel. Apart from the case of Kerre and Goossens [2], Caroli and colleagues [12] describe a surgical nurse who showed work dependent eczematous lesions on her forearms which developed after surgical gowns sterilized with EtO had been used at work. Patch testing with a piece of EtO-sterilized gown resulted in a delayed type hypersensitivity reaction at the test site; a patch test with gamma-sterilized material was negative. After EtO-sterilized gowns had been eliminated from the workplace, no relapse of her eczema occurred. Patch tests with epichlorohydrin had not been performed. 

The sensitizing potential of epoxy compounds is well known and cross reactions between different substances may occur. Sensitization to bisphenol A based epoxy resins is frequently observed in the contracting industry and may also occur among workers of epoxy resin plants. During the manufacturing process of epoxy resins, an exposure to epichlorohydrin with a subsequent sensitization may occur [5, 6]. 

The diagnosis of irritant contact dermatitis was established in 7/8 patients. All but one of the patients had a history of atopy, of previous contact dermatitis or of drug hypersensitivity (Table 1). EtO is a known strong irritant [1]. Severe postoperative burns from re-usable surgical gowns and drapes that had been sterilized with EtO and not properly ventilated were reported by Biro and colleagues [13]. EtO residues varied between 3,600 ppm and 10,800 ppm. Occupational irritant contact dermatitis to EtO was reported among 9 pharmaceutical workers who had worn EtO-sterilized overalls at work [14]. Skin lesions occuring a few hours after the use of the overalls located around the wrists with itching, redness and vesicles persisted for several weeks. Since no patch tests were performed in these individuals it remains unclear whether some of the described reactions may have been allergic. A residue of 500 ppm EtO was measured by gas chromatography in one of the overall bags. The recommended safe level for skin contact with EtO is 200 ppm [1]. EtO residues were not measured in the surgical gowns which had been used by our patients. 

Romaguera and Vilaplana [15] reported on occupational airborne contact dermatitis in nursing personnel who had to sterilize reusable hospital linen in a canister containing EtO. Similar as in our patients, 3 of the 4 patients had a personal or family background of atopy and 2 had a history of contact dermatitis. 

Apart from its sensitizing and irritant potential, EtO is known as an agent with cytotoxic, carcinogenic and mutagenic capacities. An increased frequency of leukemia and stomach cancer was reported among exposed workers [16, 17]. Therefore, medical devices sterilized with EtO should properly be aerated and whenever possible, EtO should be replaced by alternative products. 

In summary, EtO-sterilized medical devices may cause occupational irritant contact dermatitis in health personnel, but may also act as a contact allergen. We assume that the problem of occupational allergic contact dermatitis to EtO may have been underestimated in the past due to methodological difficulties in patch testing. Therefore, when allergic contact dermatitis to EtO is suspected, a patch test to 0.1% epichlorohydrin should be performed to confirm a sensitization to EtO. 

## Conflicts of interest 

None. 

**Table 1.** Table 1. Characteristics of employees with allergic or irritant contact dermatitis to ethylene oxide who were patch tested to epichlorohydrin 0.1% and 1.0%.

**Patient no.**	**Age (yrs)**	**Sex**	**Profession**	**History of atopy/contact dermatitis**	**Skin lesions**	**Patch test 0.1% epichlorohydrin**			**Patch test 1.0% epichlorohydrin**		
						24 hr	48 hr	72 hr	24 hr	48 hr	72 hr
1	38	f	Surgical nurse	Contact urticarial Contact allergy to thiuram to latex	Eczematous lesions at the wrists, spreading to the forearms Long-standing erythema Application of topical steroids	?	n.d.	+	?	n.d.	+
2	45	m	Trauma surgeon	Occupational irritant Contact dermatitis (hands)	Eczematous lesions at the wrists	–	+	–	+	+	+
3	28	m	Trauma surgeon	–	Eczematous lesions at the wrists Use of oral corticosteroids and antihistamines	?	–	–	ir	ir	ir
4	20	m	Surgical male nurse	Contact allergy to tea tree oil Family history of atopy	Eczematous lesions at the wrists						
5	24	f	Surgical nurse	Allergic rhinoconjunctivitis Drug hypersensitivity (metamizol) Family history of atopy	Eczematous lesions at the wrists	–	–	–	+	–	–
6	24	f	Surgical nurse	Atopic dermatitis Allergic asthma Contact urticaria (latex) Family history of atopy	Eczematous lesions at the wrists	–	–	–	?	?	?
7	29	f	Surgical nurse	Drug hypersensitivity (penicillin, iodinated contrast media)	Eczematous lesions at the wrists	–	–	–	?	?	?
8	23	f	Surgical nurse	Food allergy, Family history of atopy	Eczematous lesions at the wrists	–	–	–	?	?	?

?: erythema; +: erythema, infiltration, papules; ir: bulla; n.d.: not done.
